# Quality of life after low-dose rate-brachytherapy for prostate carcinoma – long-term results and literature review on QLQ-C30 and QLQ-PR25 results in published brachytherapy series

**DOI:** 10.1186/s12955-018-0844-8

**Published:** 2018-01-22

**Authors:** Daniel Buergy, Vincent Schneiberg, Joerg Schaefer, Grit Welzel, Lutz Trojan, Christian Bolenz, Frederik Wenz

**Affiliations:** 1 Department of Radiation Oncology, Universitätsmedizin Mannheim, Medical Faculty Mannheim, Heidelberg University, Mannheim, Germany; 2 Department of Urology, University Medical Center Goettingen, Göttingen, Germany; 3 Department of Urology, University of Ulm, Ulm, Germany

**Keywords:** Prostate carcinoma, Quality of life, QOL, Brachytherapy, Patient reported outcome, PRO

## Abstract

**Background:**

Patient-reported health-related quality of life (HRQOL) differs between treatment options for prostate carcinoma. Long-term HRQOL data in brachytherapy series are scarce. Therefore, we analyzed prostate-specific and general HRQOL in patients treated with brachytherapy for prostate carcinoma after long-term follow-up.

**Methods:**

Two hundred ninety-six patients with prostate carcinoma were treated with brachytherapy (01/1998–11/2003). General and prostate-specific HRQOL were measured using EORTC-QLQ-C30 and EORTC-QLQ-PR25, respectively. Patients were asked to complete the questionnaires after a median follow-up of 141 (119–181) months. QLQ-C30 results were compared to the German reference population. QLQ-PR25 results were compared to an earlier follow-up after a median of 51 months (no published QLQ-PR25 reference population for comparison). Additionally, a literature review on HRQOL data in brachytherapy series was performed.

**Results:**

One hundred six (35.8%) patients were lost to follow-up, 70 (23.6%) had died. 120 (40.5%) patients were contacted. 80 questionnaires were returned (27% of the original cohort; 91% of alive patients were ≥70 years). Sexual activity declined over time (mean scores: 40.5 vs. 45.5; *p* = 0.006), hormonal treatment-related symptoms, problems associated with incontinence aids, and burden of obstructive urinary symptoms did not differ significantly compared to the 51-month follow-up. General HRQOL was numerically better in our cohort as compared to the German reference population (> 16% relative difference for both age strata; < 70 and ≥70 years).

**Conclusions:**

Our results indicate that symptom-burden after long-term follow-up and associated prostate-specific HRQOL remains relatively stable from 51 to 141 months. General HRQOL in surviving patients was numerically better compared to the reference population.

**Electronic supplementary material:**

The online version of this article (10.1186/s12955-018-0844-8) contains supplementary material, which is available to authorized users.

## Background

There is still a lack of evidence of the optimal treatment option for men with localized prostate carcinoma [1–4]. Treatment decisions might therefore be influenced by anticipated long-term changes in quality of life. Recently, the 10-year outcomes of the ProtecT trial, comparing radical prostatectomy with external beam radiation therapy (EBRT) or active surveillance were published [4]. Cancer-specific mortality was low in all study arms (< 2%). Health-related quality of life (HRQOL) was evaluated up to 5 years after treatment [5]. Specific function scales showed that radical prostatectomy had the greatest negative effects on sexual function and urinary continence. EBRT had a temporary impact on sexual function, and negative effects on bowel function [5]. The Prostate Cancer Outcomes Study [6] showed similar results after a follow-up of 2.5 years; however, differences between the groups decreased over time. Taken together, the Prostate Cancer Outcomes Study and the ProtecT study indicate a need for long-term quality of life assessments because toxicity and specific HRQOL change over time. Unfortunately, both studies did not have a brachytherapy arm.

Brachytherapy for localized prostate carcinoma is a well-established treatment option for localized prostate carcinoma in the US [1] and the EU [2, 3]. Although HRQOL has been reported in brachytherapy series, long-term results are scarce. The most detailed analyses have been reported by Miller [7], Pardo [8], and Sanda [9].

Miller et al. [7] analyzed quality of life after a median follow-up of up to 6.2 years after LDR-brachytherapy. The authors compared the results to an earlier analysis at a follow-up of 2.6 years. Irritative-obstructive symptoms and bowel symptoms improved over time (2.6 vs. 6.2 years). Patient-reported urinary incontinence symptoms worsened. Pardo and co-workers [8] compared patients treated with radical prostatectomy, EBRT or brachytherapy after 3 years. They reported that: “long-term modifications of adverse effects (…) tended to reduce differences between treatment groups (radical prostatectomy, EBRT, brachytherapy) over time”. Despite the reduction of differences, the initial patient-reported HRQOL profile remained characteristic for each treatment option over the observation period of 3 years. The third study with a follow-up of 2 years has been published by Sanda et al. [9]. The authors described similar HRQOL profiles compared to the aforementioned analysis.

In the German population with prostate carcinoma, the applicable guideline [3] specifically recommends the use of available European Organization for Research and Treatment of Cancer (EORTC-) tools for HRQOL evaluation in patients with prostate carcinoma. The EORTC-QLQ-C30 questionnaire [10, 11] evaluates general HRQOL and burden of typical cancer symptoms. Prostate-specific HRQOL is measured by the EORTC-QLQ-PR25 questionnaire [11, 12]. Based on the long-term observations after radical prostatectomy and EBRT, we assumed that HRQOL might change over time after brachytherapy. We decided to investigate the impact of brachytherapy on HRQOL in patients with long-term follow-up (119–141 months). Available studies on this topic are scarce. Basically, only Drummond et al., reported on a registry study which included brachytherapy patients with a follow-up of more than ten years after initial diagnosis using QLQ-PR25 and QLQ-C30. Even in this national registry analysis, the authors were only able to identify 33 brachytherapy patients with a follow-up of five to ten years and four patients with a follow-up of at least ten years. All other studies we identified in our literature research did not have a follow-up of ten years or more (see Additional file 1: Table S1 for details).

Taken together, there is a need for long-term HRQOL data in patients with prostate carcinoma.

Our study aims to fill this gap. We analyzed long-term general-, and prostate-specific HRQOL as recommended by the EORTC in a single-center German patient cohort treated with LDR brachytherapy. Our patient cohort has already been analyzed by Schaefer et al. after a follow-up of 51 months (data cut-off: 2004) [13]. The authors reported on 231 surveys from 296 patients who were initially treated, 12.8% (*n* = 38) had died before 2004 (*n* = 258 alive) and 9.1% (*n* = 27) patients did not return evaluable surveys. Urinary symptom scores showed a mean score of 29.7 (QLQ-PR25); 46.7% of patients reported moderate to strong urination frequency symptoms. Incontinence aids were required by 12.9% of patients. Severe erectile dysfunction was described by 39.6% of patients. Fecal incontinence was rare (2.8% moderate-severe). These data are in line with available literature. [7–9, 14]. Based on the trends identified by Miller et al., we assumed that irritative-obstructive symptoms might further improve over time while urinary incontinence burden was expected to worsen over time [7].

General HRQOL was not evaluated in our cohort at an earlier time point. General HRQOL results were therefore compared to the German age-stratified reference population.

Prostate-specific HRQOL was compared to the 1st follow-up (51 months). An eligible reference population is currently not available for QLQ-PR25.

Furthermore, we performed a literature review to identify other studies analyzing QLQ-C30 and/or QLQ-PR25 results after long-term follow-up of prostate carcinoma patients treated with brachytherapy in international patient populations.

## Methods

*Patients:* The study was approved by the ethics committee of Heidelberg University, Medical Faculty Mannheim (2013-597 N-MA).

From January 1998 to November 2003, a total of 296 patients underwent Iodine-125 seed implantation as monotherapy for localized prostate carcinoma. The brachytherapy planning target volume consisted of the prostate gland with a 5 mm anterior and lateral margin on each ultrasound slice. The minimal peripheral dose was > 140 Gy for Iodine-125 seeds. Intraoperative planning using ultrasound imaging was performed with treatment planning software (Variseeds, Varian, Charlottesville, VA, USA). Occasionally, pretreatment planning was also performed. 6 weeks post implant. All patients received post-planning CT evaluations at 6 weeks following seed placement. Antihormone therapy was administered at the urologist’s discretion in case of biochemical recurrence but was not administered routinely prior (or concurrently) with brachytherapy.

Further details on the treated patient cohort and earlier QLQ-PR25 measurements have been published by Schaefer et al. [13]. After a median follow-up of 51 months (data cut-off 12/2004), 38 patients had died, 258 patients were alive and 231 patients had returned questionnaires.

HRQOL as measured by QLQ-C30 has not been assessed previously in this patient group. Baseline HRQOL results were also not available. Patients of the original cohort were contacted by phone, and, if not available at repeated calls, via relatives, general practitioners or urologists. Questionnaires were sent via regular mail to all patients of the original cohort who were alive and could be identified.

*Questionnaires:* EORTC QLQ-C30 (V 3.0) includes five functional scales: physical, role, cognitive, emotional, and social function. Three symptom scales are evaluated: fatigue, nausea/vomiting and pain. Furthermore it includes a general health status/global HRQOL scale and a number of single items assessing symptoms commonly reported by cancer patients: Dyspnoea, loss of appetite, insomnia, constipation, diarrhea, and (perceived) financial impact of the disease [10, 11].

Validity and reliability of the QLQ-C30 have been extensively reported in a variety of treatment settings, including prostate carcinoma [10, 12, 15]. As described previously, differences in scores of QLQ-C30 scales were considered clinically relevant when the difference exceeded 10 points [10, 14].

QLQ-PR25, EORTC’s prostate specific module incorporates 25 items and has been designed and validated for localized and metastatic prostate cancer [11, 12]. The latest version of the module includes 3 multi-item symptom scales: Urinary-, bowel-, and hormonal treatment-related symptoms, respectively. Furthermore, it includes a single item to measure *“bother due to the use of an incontinence aid”* (“incontinence burden”) which is conditionally applied if patients are wearing an incontinence aid. Sexual activity is incorporated as a function scale. In case of any sexual activity during the last 4 weeks, the sexual functioning scale is applied as a conditional functioning scale [12].

All of the scales and single-item measures range in score from 0 to 100. A high score for a functional scale represents a high/healthy level of functioning. The same is true for the global HRQOL. A high score for a symptom scale represents a high level of symptomatology/problems [11].

*Search strategy:* To identify comparable datasets with long-term follow-up after brachytherapy, we queried PubMed with the following search terms: *(qlq-c30 OR c30 OR qlqc30) AND brachytherapy AND prostate*. Search criteria for QLQ-PR25 were as follows: *(qlq-pr25 OR pr25 OR qlqpr25) AND brachytherapy*. All articles in languages other than English were excluded from further evaluation.

All statistical calculations were performed using SPSS (V 15.0), or “R”, a language and environment for statistical computing that is available for free online [16]. Scoring of scales was computed with a modified version of an R package published by Anota et al. [17] Spearman’s nonparametric correlation coefficient was calculated to identify correlations between nonparametric variables. Differences in QOL variables over time were calculated using Wilcoxon’s approach for paired samples; in case of dichotomous variables, McNemars test was used to compute statistical differences. The Mann-Whitney *U* test was used to calculate significance of differences between independent samples. Both approaches for paired analysis include only patients who were available at both time points of the analysis (i.e. patients who were not available at the 2nd analysis are excluded in the first dataset) and show differences for paired samples of the same patient at different time points. If not otherwise specified, all mean values detailed in the results section apply to all patients who returned the required item at a certain time point.

## Results

Out of 258 patients who were alive in 2004, 106 were lost to follow-up or could not be reached (no information: *n* = 100; clinical information available but patients could not be reached: *n* = 6). 32 patients had died since the last follow-up in 2004 (38 + 32 = 70 patients diseased). All available clinical data are summarized in Table 1. The remaining 120 patients were contacted via phone. Fourteen patients declined to participate. Evaluable questionnaires were returned from 80 patients. Mean age of patients who answered the questionnaires was 78.1 years (range: 63–94; 7 patients were 60–69 years old, all others were ≥70 years old). Subgroup analysis were performed (age groups: 70–74, 75–79, 80–84, 85 or older) but due to limited patient numbers, all patients ≥70 were considered elderly which is in line with the German reference population analysis [18]. The median period of follow-up after implantation was 141 months (range: 119–181 months). Patients who returned evaluable questionnaires had low, intermediate, and high risk prostate carcinoma in 62.5%, 10%, and 5%, respectively as classified by D’Amico et al. [19]. Staging was incomplete or missing in 22.5% of responders. Further clinical characteristics and outcome data on the full patient cohort are summarized in Table 1. We have not performed a detailed evaluation of clinical outcomes.

**Table 1:** Characteristics and outcome of patients treated with brachytherapy

Characteristics		Initial Cohort *n* = 296 (100%)
Gleason Score	≤ 6	173 (58.4%)
	> 6	32 (10.8%)
	Unknown	91 (30.7%)
Preoperative PSA value	≤ 10 ng/ml	208 (70.3%)
	> 10 (−20) ng/ml	62 (20.9%)
	> 20 ng/ml	26 (8.8%)
Risk group^a^	Low	110 (37.1%)
	Intermediate	26 (8.8%)
	High	52 (17.6%)
	Unknown	90 (30.4%)
	T3 (*n* = 15), N1 (*n* = 2), or M1 (*n* = 1)	18 (6.1%)
Endpoint	Clinical outcome	Number of Patients (Percentage)
	All patients	296 (100%)
Overall survival in all (*n* = 296) patients	Died before 2004 (Original cohort) Died between 2004 and 2013 (Current cohort)	38 (12.8%) 32 (10.8%)
	Unknown Alive but not willing to participate Alive and contacted by phone No available contact information but alive; clinical information as reported by urologist or general practitioner	100 (33.8%) 6 (2%) 114 (38.5%) 6 (2%)
	All deaths	70 (100%)
Causes of death in 70 patients who had died.	Prostate carcinoma Unrelated cancer *Leukemia* *Gastric cancer* *Pancreatic cancer* *Lung cancer* Cardiovascular^b^ Infection^c^ Kidney failure Other or unknown	20 (28.6%) 8 (11.4%) *2* *1* *3* *2* 16 (22.9%) 3 (4.3%) 3 (4.3%) 20 (28.6%)
	Patients contacted	120 (100%)
Biochemical recurrence at any time in *n* = 120 patients with clinical information who were alive at final follow-up	Biochemical recurrence present at any time	22 (18.3%)
	No biochemical recurrence	68 (56.7%)
	Unknown biochemical outcome	30 (25%)
	All patients with biochemical recurrence	22 (100%)
Clinical recurrence in patients who are alive and had biochemical recurrence	Local recurrence at any time Distant metastases at any time Distant and local recurrence Unknown	10 (45.4%) 2 (9.1%) 1 (4.5%) 13 (59.1%)

^a^Patients with unknown Gleason Score were classified as high risk, if any high risk criteria were met (e.g. patients with PSA > 20 ng/ml were considered as high risk, even in case of unknown Gleason Score)

^b^Cardiovascular includes: Myocardial infarction, stroke, cerebral hemorrhage in a Marcumar patient, ruptured aortic aneurism, and cardiac arrest in coronary heart disease

^c^Infection includes pneumonia and sepsis

*QLQ-C30 long-term results and comparison to German reference data.*

**Fig. 1:**
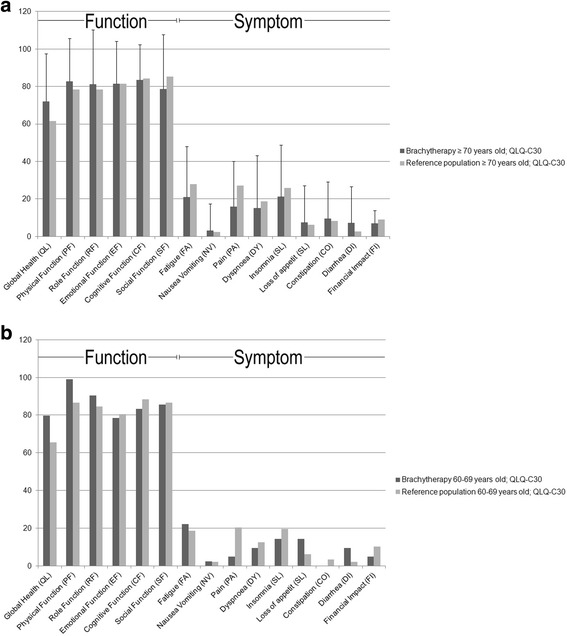
EORTC-QLQ-C30 scales after long-term follow-up in patients treated with brachytherapy compared to the German reference population. Age stratification according to Schwarz and Hinz [18], 1**a**) Population ≥ 70 years old and 1**b**) Population 60–69 years old. Higher function values indicate better function. Higher symptom values indicate worse symptoms. Bars represent mean values. Lines show standard deviations

QLQ-C30 was not scored at baseline, therefore, we compared our long-term results to the German reference population [18]. Age groups were stratified as described by Schwarz et al. (60–69 years vs. ≥ 70 years). Only 7 patients were left in the younger (60–69 years) group in our dataset, therefore the following results refer to the elderly group of patients (≥ 70 years). All data of both the young (60–69), and the elderly (≥ 70 years) patients as compared to the German stratified reference population are summarized in Fig. 1 [18].

Global quality of life was increased (i.e. better function) by 16.9% in our elderly study group as compared to the German reference population (10.4 points difference in the elderly group, 14.2 points difference in the younger group).

Physical, and role function scales were also increased but not clinically relevant as defined by the EORTC [10].

Emotional function results were similar (81.4 vs. 81.5), social function was decreased but the difference was not clinically relevant (6.6 points).

The overall pain item score was lower in our group (40.7%; 11 points difference). Furthermore, dyspnoea, insomnia, and financial impact scores were lower compared to the reference population (difference below threshold for clinical relevance).

Elevated symptom scores in the brachytherapy group were as follows: diarrhea, constipation, and appetite loss, all of these, differences were too small to be clinically relevant as defined by the EORTC; (see Fig. 1 for details).

*Changes in EORTC-QLQ-PR25 since last follow-up and identification of other long-term data in the literature for comparison (see* Table 2 *for details).*

**Table 2:** Mean QLQ-PR25 values of the brachytherapy group after 51 months compared to the long-term follow-up after 141 months. There are two columns for the first follow-up, the first one shows only the (4.3-year) results for patients alive after long-term follow-up; the second shows the results for all patients

Item or scale	Follow-up 4.3 years; only long-term survivors shown	Follow-up 4.3 years all patients shown	Follow-up 11.7 years
Urinary-related symptoms	25.6 (SD = 19.1; *n* = 79)	29.7 (SD = 23.7; *n* = 240)	24.3 (SD = 21.8; n = 79)
Bowel-related symptoms	7.3 (SD = 14.1; *n* = 74)	8.2 (SD = 13.4; *n* = 223)	8.5 (SD = 12.3; *n* = 73)
Hormone treatment-related symptoms	11.2 (SD = 11.6; n = 74)	15.5 (SD = 16.1; *n* = 230)	14.7 (SD = 16.0; *n* = 76)
Sexual activity	51.1 (SD = 29.1; n = 76)	45.5 (SD = 32.4; *n* = 234)	40.5 (SD = 31.2; n = 74)
Sexual function	61.6 (SD = 19.3; *n* = 58)	57.5 (SD = 23.7; *n* = 154)	64.5 (SD = 23.1; *n* = 34)
Bother due to use of an incontinence aid	50 (SD = 45.9; n = 6)	43.4 (SD = 38.6; *n* = 33)	66.7 (SD = 36; *n* = 13)
Incontinence^a^	7.5%	12.9%	16.3%

^a^Estimated by percentage of patients wearing an incontinence aid

Sexual activity was significantly lower in the long-term follow-up as compared to 2004 (*p* = 0.006; mean scores: 40.5 vs. 45.5). Over time, patients had less sex at the 2nd observation compared to the first follow-up in 2004 (*p* = 0.041; 63% vs. 58.7%). In patients who were still sexually active, sexual function did not differ significantly between 2004 and the current follow-up (*p* > 0.05; 57.5 vs. 64.5). Patient’s age was significantly associated with reduced sexual activity at both the 2004, and the current analysis (*r* = − 0.341, *p* < 0.001; and *r* = − 0.288, *p* = 0.013). Urinary symptoms numerically improved by 18.2% during long-term follow-up, however the difference was not statistically significant in the paired comparison (*p* > 0.05; mean urinary-related symptoms scores: 24.3 vs. 29.7). Mean hormonal treatment-related symptoms in the whole group at each time point minimally decreased over time in the whole 2004 vs. current group (15.5 vs. 14.7). Nevertheless, in patients who were alive and eligible at both observations, the score increased over time (i.e. the patients who stayed alive and were available at the 1st and the 2nd analysis had an increase in their hormonal treatment-related symptoms score over time; p = 0.013, 11.2 vs. 14.7).

The percentage of patients who required an incontinence aid was 12.9% in the whole group of 2004 and increased slightly to 16.3% in 2013. Problems associated with incontinence aids increased numerically over time but the difference was not significant, probably due to the limited number of patients who needed an incontinence aid (mean incontinence burden 43.4 vs. 66.7; *p* > 0.05). Bowel symptoms were low at any time point and did not differ significantly over time (p > 0.05; mean 8.2 vs. 8.5). Table 2 shows all QLQ-PR25 results over time. There is no German reference population to which we could compare our results.

Results of our systematic literature search for other long-term QOL results using EORTC QLQ-PR25 in patients receiving brachytherapy are shown in Additional file 1: Table S1. We were not able to identify a similar cohort with a long-term follow-up. The only study including (*n* = 4) patients with more than 10 years of follow-up was the aforementioned Irish registry analysis [20].

*Associations of local symptom burden as measured by QLQ-PR25 with general Quality of life.*

Prostate-specific QLQ-PR25 symptom scales were negatively correlated with general quality of life (significant for incontinence burden, urinary-related, bowel-related, and hormonal treatment-related symptoms, all *p* < 0.05; see Additional file 2: Table S2 for details). Sexual activity was positively associated with global HRQOL (*p* = 0.006). Sexual function showed no correlation with global HRQOL, however this was expected as sexual function is a conditional scale which is only filled out by patients who had sexual activity during the last four weeks (i.e. patients who returned a low score still had more sexual activity as compared to patients who did not answer the questions at all). If patients without sexual activity were compared to those with (any) sexual activity, global HRQOL was increased in those who still had sex (mean global HRQOL scores: 65.6 vs. 77.1; *p* = 0.041). Patients who needed to wear incontinence aids showed reduced global HRQOL scores as compared to patients who did not require an incontinence aid (global HRQOL 75 vs. 55.1). All associations of QLQ-PR25 scores with general quality of life as measured by global HRQOL are shown in Additional file 2: Table S2.

## Discussion

Although some studies on prostate carcinoma patients cover long-term follow-up periods well above 10 years [21], they rarely include patients treated with brachytherapy [22]. Most recently, two studies on side effect profiles (without HRQOL) in brachytherapy series have been published: Cosset et al. [22] reported on a French series of patients who had received LDR brachytherapy with a median follow-up of ~ 11 years. Toxicity, according to NCI-CTC (2.0) [23] but not quality of life was assessed. The authors reported a cumulative rate of grade 3–4 chronic urinary toxicity of 5.8% and a low incidence of rectal toxicity (grade 3–4: 1.65%). Grade 3 erectile toxicity was observed in 22.1% of patients, however in the elderly patient group (> 70 years at treatment), the rate was 41% [22].

Another study with a long-term toxicity assessment in a population with young prostate carcinoma patients (≤ 60 years at treatment) has been published by Buckstein et al. [24], similar to the French study, the authors found low rates of GU toxicities (4.5% continued grade ≥ 2 toxicity after 10 years), and no GI toxicity persisted after 10 years (cumulative: 8.3% grade ≥ 2 GI events were observed). Of patients who were potent before treatment, 69% remained so after 10 years of follow-up [24]. While these studies indicate a favorable long-term safety profile, it has been shown that HRQOL results might differ in a clinically relevant and statistically significant way from physician-assessed side effects [25–27]. This has been specifically observed in prostate carcinoma by Gravis et al. [25] in the randomized setting of the GETUG-AFU 15 trial. HRQOL data showed worse side effects as perceived by the patients when compared to physician’s assessment.

Patient-reported HRQOL results should therefore be analyzed for each treatment option and results must be discussed with patients when it comes to treatment decisions. In our literature search, we were able to identify several brachytherapy studies which reported HRQOL as proposed by the EORTC. However we could not identify a study with a long-term follow up (median ≥ 10 years) that reported QLQ-C30, or QLQ-PR25 results.

Data from our patient series indicate that patients who received brachytherapy for localized prostate carcinoma have a high general quality of life after long-term follow-up. Surprisingly, global HRQOL scores were well above the German age-adjusted reference population [18]. Furthermore, our cohort of patients had less pain as compared to the reference. Social function as measured by SF was worse in our population although the difference would not be considered clinically relevant (6.6 points) [10, 14]. All other general HRQOL scores showed minor differences as compared to the reference population. Multiple studies have shown that prostate carcinoma patients may have similar or even better general HRQOL results after local treatment compared to healthy populations [7, 28–30]. This observation has been explained as “posttraumatic growth” or “benefit finding”, i.e. idenfication of a benefit from adversity [28, 31–33].

In contrast to general quality of life, there is no German reference dataset concerning prostate-specific quality of life as measured by QLQ-PR25. Due to the lack of such a reference population, we compared our dataset to the first available follow-up visit. The percentage of patients who were still sexually active was only slightly reduced as compared to the first follow-up (58.7 vs. 63%), however, patient’s perceived quantity and quality of sexual intercourses was significantly reduced. In patients who were still sexually active, sexual function had not decreased over time. As expected, age was significantly associated with lower sexual activity scores in both analyses. Surprisingly, urinary function as measured by urinary-related symptoms improved over time; however the difference was not statistically significant (scores: 24.3 vs. 29.7). The Improvement of urinary urgency has been observed in other studies during the first years post-therapy [34]. It is currently uncertain how long this trend continues and when it is reversed by the onset of age-related urinary symptoms. We did not evaluate systemic treatments or clinical outcomes systematically, so it is unclear if the increased symptom burden in hormonal treatment-related symptoms was age-related or related to hormonal therapy in case of cancer recurrence. Bowel symptoms did not change over time in our patient cohort. The percentage of patients with incontinence numerically increased by 3.4%. Additionally, problems associated with incontinence aids significantly increased in the long-term follow-up and general HRQOL was clinically meaningful and significantly reduced in case of incontinence. Our study has several shortcomings:

1. It is a retrospectively designed study which is prone to bias. Specifically, patients may be more likely to answer questionnaires, when they are relatively healthy as opposed to unfit patients. This might, to some extent, explain the good outcome of our study cohort and it underscores the need for prospective studies.
2. We are lacking pre-treatment data in both QLQ-C30, and QLQ-PR25 questionnaires, therefore we are not able to analyze changes from baseline. Furthermore, we analyzed no QLQ-C30 data in the first (post-treatment) analysis; hence differences over time cannot be assessed.
3. We did not prospectively evaluate all treatment outcomes in detail. Therefore, we were not able to show associations between treatment outcomes or salvage treatments and HRQOL.
4. Our initial population included patients who were unfit for other treatments and patients who decided to undergo brachytherapy even if they were not optimal candidates. This resulted in 17.6% of patients with high-risk, and 6.1% of patients with advanced disease (30.4% incomplete data). Our HRQOL results might be negatively influenced by this treatment selection.

Despite these shortcomings, our study provides insights for patients and caregivers about long-term HRQOL after LDR brachytherapy for prostate carcinoma.

## Conclusions

Taken together, our study shows that general HRQOL in patients treated with prostate brachytherapy is good. This is despite to a decrease in sexual function and increasing problems with incontinence (aids) over time, as well as constant bowel symptoms in 8% of patients. Total rates of incontinence in the cohort were still low (16%) and had increased only slightly (3%) over time. Both incontinence and impotence affected general quality of life in this cohort of elderly patients. Urinary-related symptoms did not worsen over time from the 5th to the 12th year of follow-up.

## Additional files

Additional file 1: Table S1. Clinical studies listed in PubMed on EORTC QLQ-C30 or QLQ-PR25 in prostate carcinoma treated with brachytherapy. (DOCX 51 kb)

Additional file 2: Table S2. Correlations between QLQ-PR25 scores and general quality of life. (DOCX 17 kb)

## References

[1] NCCN Clinical Practice Guidelines in Oncology (NCCN Guidelines®) [https://www.nccn.org/professionals/physician_gls/pdf/prostate.pdf].

[2] Heidenreich A, Bastian PJ, Bellmunt J, Bolla M, Joniau S, van der Kwast T, Mason M, Matveev V, Wiegel T, Zattoni F (2014). EAU guidelines on prostate cancer. Part 1: screening, diagnosis, and local treatment with curative intent-update 2013. Eur Urol.

[3] Leitlinienprogramm Onkologie (Deutsche Krebsgesellschaft, Deutsche Krebshilfe, AWMF): Interdisziplinaere Leitlinie der Qualitaet S3 zur Frueherkennung, Diagnose und Therapie der verschiedenen Stadien des Prostatakarzinoms [http://www.leitlinienprogramm-onkologie.de/leitlinien/prostatakarzinom/].

[4] Hamdy FC, Donovan JL, Lane JA, Mason M, Metcalfe C, Holding P, Davis M, Peters TJ, Turner EL, Martin RM (2016). 10-year outcomes after monitoring, surgery, or radiotherapy for localized prostate cancer. N Engl J Med.

[5] Donovan JL, Hamdy FC, Lane JA, Mason M, Metcalfe C, Walsh E, Blazeby JM, Peters TJ, Holding P, Bonnington S (2016). Patient-reported outcomes after monitoring, surgery, or radiotherapy for prostate cancer. N Engl J Med.

[6] Resnick MJ, Koyama T, Fan KH, Albertsen PC, Goodman M, Hamilton AS, Hoffman RM, Potosky AL, Stanford JL, Stroup AM (2013). Long-term functional outcomes after treatment for localized prostate cancer. N Engl J Med.

[7] Miller DC, Sanda MG, Dunn RL, Montie JE, Pimentel H, Sandler HM, McLaughlin WP, Wei JT (2005). Long-term outcomes among localized prostate cancer survivors: health-related quality-of-life changes after radical prostatectomy, external radiation, and brachytherapy. J Clin Oncol.

[8] Pardo Y, Guedea F, Aguilo F, Fernandez P, Macias V, Marino A, Hervas A, Herruzo I, Ortiz MJ, Ponce de Leon J (2010). Quality-of-life impact of primary treatments for localized prostate cancer in patients without hormonal treatment. J Clin Oncol.

[9] Sanda MG, Dunn RL, Michalski J, Sandler HM, Northouse L, Hembroff L, Lin X, Greenfield TK, Litwin MS, Saigal CS (2008). Quality of life and satisfaction with outcome among prostate-cancer survivors. N Engl J Med.

[10] Aaronson NK, Ahmedzai S, Bergman B, Bullinger M, Cull A, Duez NJ, Filiberti A, Flechtner H, Fleishman SB, de Haes JC (1993). The European Organization for Research and Treatment of cancer QLQ-C30: a quality-of-life instrument for use in international clinical trials in oncology. J Natl Cancer Inst.

[11] Fayers P, Aaronson N, Bjordal K, Groenvold M, Curran D, Bottomley A, Group obotEQoL. *The EORTC QLQ-C30 Scoring Manual (3*^*rd*^*Edition).* Brussels: European organisation for research and treatment of. Cancer. 2001;

[12] van Andel G, Bottomley A, Fossa SD, Efficace F, Coens C, Guerif S, Kynaston H, Gontero P, Thalmann G, Akdas A (2008). An international field study of the EORTC QLQ-PR25: a questionnaire for assessing the health-related quality of life of patients with prostate cancer. Eur J Cancer.

[13] Schafer JW, Welzel G, Trojan L, Eppler N, Harrer K, Michel MS, Alken P, Wenz F (2008). Long-term health-related quality-of-life outcomes after permanent prostate brachytherapy. Onkologie.

[14] Boettcher M, Haselhuhn A, Jakse G, Brehmer B, Kirschner-Hermanns R (2012). Overactive bladder syndrome: an underestimated long-term problem after treatment of patients with localized prostate cancer?. BJU Int.

[15] Barry LC, Kasl SV, Lichtman J, Vaccarino V, Krumholz HM (2006). Social support and change in health-related quality of life 6 months after coronary artery bypass grafting. J Psychosom Res.

[16] R_Core_Team: R: A language and environment for statistical computing.: R foundation for Statistical Computing, Vienna, Austria.; 2015.

[17] QoLR: Analysis of Health-Related Quality of Life in Oncology [https://cran.r-project.org/web/packages/QoLR/index.html].

[18] Schwarz R, Hinz A (2001). Reference data for the quality of life questionnaire EORTC QLQ-C30 in the general German population. Eur J Cancer.

[19] AV D’A, Whittington R, Malkowicz SB, Schultz D, Blank K, Broderick GA, Tomaszewski JE, Renshaw AA, Kaplan I, Beard CJ, Wein A (1998). Biochemical outcome after radical prostatectomy, external beam radiation therapy, or interstitial radiation therapy for clinically localized prostate cancer. JAMA.

[20] Drummond FJ, Kinnear H, O'Leary E, Donnelly GA, Sharp L (2015). Long-term health-related quality of life of prostate cancer survivors varies by primary treatment. Results from the PiCTure (prostate cancer treatment, your experience) study. J Cancer Surviv.

[21] Popiolek M, Rider JR, Andren O, Andersson SO, Holmberg L, Adami HO, Johansson JE (2013). Natural history of early, localized prostate cancer: a final report from three decades of follow-up. Eur Urol.

[22] Cosset JM, Flam T, Belin L, Thiounn N, Pierrat N, Pontvert D, Wakil G, Savignoni A, Chauveinc L (2016). Long-term results of permanent implant prostate cancer brachytherapy: a single-institution study of 675 patients treated between 1999 and 2003. Cancer Radiother.

[23] Trotti A, Byhardt R, Stetz J, Gwede C, Corn B, Fu K, Gunderson L, McCormick B, Morrisintegral M, Rich T (2000). Common toxicity criteria: version 2.0. An improved reference for grading the acute effects of cancer treatment: impact on radiotherapy. Int J Radiat Oncol Biol Phys.

[24] Buckstein M, Carpenter TJ, Stone NN, Stock RG (2013). Long-term outcomes and toxicity in patients treated with brachytherapy for prostate adenocarcinoma younger than 60 years of age at treatment with minimum 10 years of follow-up. Urology.

[25] Gravis G, Marino P, Joly F, Oudard S, Priou F, Esterni B, Latorzeff I, Delva R, Krakowski I, Laguerre B (2014). Patients' self-assessment versus investigators' evaluation in a phase III trial in non-castrate metastatic prostate cancer (GETUG-AFU 15). Eur J Cancer.

[26] Petersen MA, Larsen H, Pedersen L, Sonne N, Groenvold M (2006). Assessing health-related quality of life in palliative care: comparing patient and physician assessments. Eur J Cancer.

[27] Laugsand EA, Sprangers MA, Bjordal K, Skorpen F, Kaasa S, Klepstad P (2010). Health care providers underestimate symptom intensities of cancer patients: a multicenter European study. Health Qual Life Outcomes.

[28] Mols F, van de Poll-Franse LV, Vingerhoets AJ, Hendrikx A, Aaronson NK, Houterman S, Coebergh JW, Essink-Bot ML (2006). Long-term quality of life among Dutch prostate cancer survivors: results of a population-based study. Cancer.

[29] Clark JA, Inui TS, Silliman RA, Bokhour BG, Krasnow SH, Robinson RA, Spaulding M, Talcott JA (2003). Patients' perceptions of quality of life after treatment for early prostate cancer. J Clin Oncol.

[30] Korfage IJ, Essink-Bot ML, Borsboom GJ, Madalinska JB, Kirkels WJ, Habbema JD, Schroder FH, de Koning HJ (2005). Five-year follow-up of health-related quality of life after primary treatment of localized prostate cancer. Int J Cancer.

[31] Bellizzi KM, Blank TO (2006). After prostate cancer: predictors of well-being among long-term prostate cancer survivors. Cancer.

[32] Schulz U, Mohamed NE (2004). Turning the tide: benefit finding after cancer surgery. Soc Sci Med.

[33] Calhoun LG, Cann A, Tedeschi RG, McMillan J (2000). A correlational test of the relationship between posttraumatic growth, religion, and cognitive processing. J Trauma Stress.

[34] Buron C, Le Vu B, Cosset JM, Pommier P, Peiffert D, Delannes M, Flam T, Guerif S, Salem N, Chauveinc L, Livartowski A (2007). Brachytherapy versus prostatectomy in localized prostate cancer: results of a French multicenter prospective medico-economic study. Int J Radiat Oncol Biol Phys.

