# Supplementary material for: Quality of life after low-dose rate-brachytherapy for prostate carcinoma – long-term results and literature review on QLQ-C30 and QLQ-PR25 results in published brachytherapy series
Source: Health Qual Life Outcomes. 2018 Jan 22;16:21. doi: 10.1186/s12955-018-0844-8 (PMC5778674; doi:10.1186/s12955-018-0844-8)
Supplement: Supplementary file 1 — Clinical studies listed in PubMed on EORTC QLQ-C30 or QLQ-PR25 in prostate carcinoma treated with brachytherapy. (DOCX 51 kb) [file 12955_2018_844_MOESM1_ESM.docx]

**Supplementary Table 1:** Clinical studies listed in PubMed on EORTC QLQ-C30 or QLQ-PR25 in prostate carcinoma treated with brachytherapy

| **1^st^ author of study, year of publication** | **Technique** | **Follow-up** | **EORTC Questionnaire** | **Location** |
| --- | --- | --- | --- | --- |
| Acar, 2014[1] | Not specified if HDR or LDR, registry study vs. AS vs. RP | Median 40 months | QLQ-C30, QLQ-PR25 | The Netherlands |
| Aluwini, 2015[2] | HDR | Mean: 35 months | QLQ-PR25 | The Netherlands |
| Boettcher, 2011[3] | HDR and LDR (Iodine-125) vs. RP | Baseline up to 36 months | QLQ-C30 | Germany |
| Buron, 2006[4] | Iodine-125, LDR vs. RP | 2 years after treatment | QLQ-C30, QLQ-PR25 | France |
| Conaglen, 2013[5] | HDR and EBRT | Baseline to 2 years | QLQ-C30, QLQ-PR25 | New Zealand |
| Denham, 2012[6] | HDR as potential boost after EBRT +/- adjuvant leuprorelin | Baseline to 36 months | QLQ-PR25 | Australia and New Zealand  (prospective TROG 03.04 trial) |
| Drummond, 2012[7] | RP, EBRT, brachytherapy (not defined if HDR or LDR) | 2-18 years; brachytherapy series: < 5 years: 70 %; 5-10 years: 27 %; > 10 years: 3 % | QLQ-C30, QLQ-PR25 | Ireland; patients were identified via The National Cancer Registry in Ireland. |
| Eisenmann, 2015[8] | Not specified if HDR or LDR; brachytherapy +/- EBRT vs. RP | Baseline up to 24 months | QLQ-C30 | Germany |
| Emara, 2012[9] | Iodine-125, LDR | 5-10 years | Only bowel syndromes were scored using QLQ-C30 and QLQ-PR25; all other symptoms: IPSS | UK |
| Evers, 2010[10] | Iodine-125, LDR +/- NHT | Baseline to 1 year | QLQ-C30, QLQ-PR25 | The Netherlands |
| Galalae, 2004[11] | HDR | Median 6.5 years | QLQ-C30 | Germany |
| Giberti, 2009[12] | Iodine-125, LDR vs. RP | 6 months up to 5 years of mean follow-up | QLQ-C30, QLQ-PR25 | Italy |
| Hjälm-Eriksson, 2015[13] | RP vs. HDR as boost after EBRT | Mean: 7 years | QLQ-C30, QLQ-PR25 | Sweden |
| Jongkamp, 2012[14] | Iodine-125, LDR | Baseline up to 1 year; other [depression specific] items were measured up to 8 years after treatment. | QLQ-C30 (+ 3), QLQ-PR25 | The Netherlands |
| Joly, 1998[15] | HDR combined with EBRT | Mean: 4 years | QLQ-C30 | France |
| Mols, 2009[16] | Iodine-125, LDR | Baseline up to 3 months | QLQ-C30, QLQ-PR25 | The Netherlands |
| Peters, 2014[17] | Iodine-125, LDR | Baseline up to a median follow-up of 3 years | QLQ-C30 (+ 3), QLQ-PR25 | The Netherlands |
| Pettersson, 2012[18] and 2014[19] | HDR as potential boost after EBRT +/- dietary interventions | Baseline up to 2 years | QLQ-C30, QLQ-PR25 | Sweden |
| Roeloffzen, 2010[20, 21] | Iodine-125, LDR | Baseline up to 6 years | QLQ-C30, QLQ-PR25 | USA |
| Vordermark, 2006[22] | HDR as potential boost after EBRT | Mean: 14 months after BT | QLQ-C30, QLQ-PR25 | Germany |
| Vordermark, 2008[23] | Iodine-125, LDR | Baseline up to 1 year | QLQ-C30, QLQ-PR25 | Germany |
| Wahlgren, 2004[24] and 2007[25] | HDR as a boost after EBRT | 7, 24, and 77 months after BT | QLQ-C30, QLQ-PR25 | Sweden |
| Wahlgren, 2011[26] | HDR as a boost after EBRT | 70-88 months after BT | QLQ-C30 | Sweden |
| Wyler, 2009[27] | LDR (Iodine-125) vs. RP | Mean follow-up: 24 months | QLQ-C30 | Switzerland |

AS: Active Surveillance; LDR: low-dose rate brachytherapy; RP: radical prostatectomy; EBRT: External Beam Radiation Therapy; IPSS: International Prostate Symptom Score;

NHT: neoadjuvant hormonal therapy; HDR: high-dose rate brachytherapy

[1] Acar C, Schoffelmeer CC, Tillier C, de Blok W, van Muilekom E, van der Poel HG. Quality of life in patients with low-risk prostate cancer. A comparative retrospective study: brachytherapy versus robot-assisted laparoscopic prostatectomy versus active surveillance. Journal of endourology / Endourological Society. 2014;28:117-24.

[2] Aluwini S, Busser WM, Alemayehu WG, Boormans JL, Kirkels WJ, Jansen PP, et al. Toxicity and quality of life after high-dose-rate brachytherapy as monotherapy for low- and intermediate-risk prostate cancer. Radiother Oncol. 2015;117:252-7.

[3] Boettcher M, Haselhuhn A, Jakse G, Brehmer B, Kirschner-Hermanns R. Overactive bladder syndrome: an underestimated long-term problem after treatment of patients with localized prostate cancer? BJU international. 2012;109:1824-30.

[4] Buron C, Le Vu B, Cosset JM, Pommier P, Peiffert D, Delannes M, et al. Brachytherapy versus prostatectomy in localized prostate cancer: results of a French multicenter prospective medico-economic study. Int J Radiat Oncol Biol Phys. 2007;67:812-22.

[5] Conaglen HM, de Jong D, Hartopeanu C, Conaglen JV, Tyrie LK. The effect of high dose rate brachytherapy in combination with external beam radiotherapy on men's health-related quality of life and sexual function over a 2 year time span. Clin Oncol (R Coll Radiol). 2013;25:197-204.

[6] Denham JW, Wilcox C, Lamb DS, Spry NA, Duchesne G, Atkinson C, et al. Rectal and urinary dysfunction in the TROG 03.04 RADAR trial for locally advanced prostate cancer. Radiother Oncol. 2012;105:184-92.

[7] Drummond FJ, Kinnear H, O'Leary E, Donnelly, Gavin A, Sharp L. Long-term health-related quality of life of prostate cancer survivors varies by primary treatment. Results from the PiCTure (Prostate Cancer Treatment, your experience) study. J Cancer Surviv. 2015;9:361-72.

[8] Tucker JM, Eisenmann JC, Howard K, Guseman EH, Yee KE, DeLaFuente K, et al. FitKids360: design, conduct, and outcomes of a stage 2 pediatric obesity program. Journal of obesity. 2014;2014:370403.

[9] Emara AM, Chadwick E, Nobes JP, Abdelbaky AM, Laing RW, Langley SE. Long-term toxicity and quality of life up to 10 years after low-dose rate brachytherapy for prostate cancer. BJU Int. 2012;109:994-1000.

[10] Evers J, Kupper N, Kessing D, Davits R, Engelen A, Poortmans P, et al. No long-term effects of hormonal therapy preceding brachytherapy on urinary function and health-related quality of life among prostate cancer patients. Urology. 2010;76:1150-6.

[11] Galalae RM, Loch T, Riemer B, Rzehak P, Kuchler T, Kimmig B, et al. Health-related quality of life measurement in long-term survivors and outcome following radical radiotherapy for localized prostate cancer. Strahlentherapie und Onkologie : Organ der Deutschen Rontgengesellschaft [et al]. 2004;180:582-9.

[12] Giberti C, Chiono L, Gallo F, Schenone M, Gastaldi E. Radical retropubic prostatectomy versus brachytherapy for low-risk prostatic cancer: a prospective study. World J Urol. 2009;27:607-12.

[13] Hjalm-Eriksson M, Lennernas B, Ullen A, Johansson H, Hugosson J, Nilsson S, et al. Long-term health-related quality of life after curative treatment for prostate cancer: a regional cross-sectional comparison of two standard treatment modalities. Int J Oncol. 2015;46:381-8.

[14] Jongkamp VG, Roeloffzen EM, Monninkhof EM, de Leeuw JR, Lycklama a Nijeholt AA, van Vulpen M. Brachytherapy for prostate cancer does not influence long-term depression rate. Brachytherapy. 2012;11:495-501.

[15] Joly F, Brune D, Couette JE, Lesaunier F, Heron JF, Peny J, et al. Health-related quality of life and sequelae in patients treated with brachytherapy and external beam irradiation for localized prostate cancer. Annals of oncology : official journal of the European Society for Medical Oncology / ESMO. 1998;9:751-7.

[16] Mols F, Stijns P, Dankaart B, Houterman S, Vingerhoets A, Hendrikx A. Health-related quality of life in I-125 prostate brachytherapy patients treated with and without volume-reducing hormone therapy: results of a short-term prospective study. J Endourol. 2009;23:153-9.

[17] Peters M, Maenhout M, van der Voort van Zyp JR, Moerland MA, Moman MR, Steuten LM, et al. Focal salvage iodine-125 brachytherapy for prostate cancer recurrences after primary radiotherapy: a retrospective study regarding toxicity, biochemical outcome and quality of life. Radiother Oncol. 2014;112:77-82.

[18] Pettersson A, Johansson B, Persson C, Berglund A, Turesson I. Effects of a dietary intervention on acute gastrointestinal side effects and other aspects of health-related quality of life: a randomized controlled trial in prostate cancer patients undergoing radiotherapy. Radiother Oncol. 2012;103:333-40.

[19] Pettersson A, Nygren P, Persson C, Berglund A, Turesson I, Johansson B. Effects of a dietary intervention on gastrointestinal symptoms after prostate cancer radiotherapy: long-term results from a randomized controlled trial. Radiother Oncol. 2014;113:240-7.

[20] Roeloffzen EM, Hinnen KA, Battermann JJ, Monninkhof EM, van Roermund JG, van Gellekom MP, et al. The impact of acute urinary retention after iodine-125 prostate brachytherapy on health-related quality of life. Int J Radiat Oncol Biol Phys. 2010;77:1322-8.

[21] Roeloffzen EM, Lips IM, van Gellekom MP, van Roermund J, Frank SJ, Battermann JJ, et al. Health-related quality of life up to six years after (125)I brachytherapy for early-stage prostate cancer. Int J Radiat Oncol Biol Phys. 2010;76:1054-60.

[22] Vordermark D, Wulf J, Markert K, Baier K, Kolbl O, Beckmann G, et al. 3-D conformal treatment of prostate cancer to 74 Gy vs. high-dose-rate brachytherapy boost: a cross-sectional quality-of-life survey. Acta Oncol. 2006;45:708-16.

[23] Vordermark D. Quality of life and satisfaction with outcome among prostate-cancer survivors. N Engl J Med. 2008;359:201; author reply -2.

[24] Wahlgren T, Brandberg Y, Haggarth L, Hellstrom M, Nilsson S. Health-related quality of life in men after treatment of localized prostate cancer with external beam radiotherapy combined with (192)ir brachytherapy: a prospective study of 93 cases using the EORTC questionnaires QLQ-C30 and QLQ-PR25. Int J Radiat Oncol Biol Phys. 2004;60:51-9.

[25] Wahlgren T, Nilsson S, Lennernas B, Brandberg Y. Promising long-term health-related quality of life after high-dose-rate brachytherapy boost for localized prostate cancer. Int J Radiat Oncol Biol Phys. 2007;69:662-70.

[26] Wahlgren T, Levitt S, Kowalski J, Nilsson S, Brandberg Y. Use of the Charlson combined comorbidity index to predict postradiotherapy quality of life for prostate cancer patients. International journal of radiation oncology, biology, physics. 2011;81:997-1004.

[27] Wyler SF, Engeler DS, Seelentag W, Ries G, Schmid HP. Health-related quality of life after radical prostatectomy and low-dose-rate brachytherapy for localized prostate cancer. Urologia internationalis. 2009;82:17-23.
