# Supplementary material for: Quality of life after low-dose rate-brachytherapy for prostate carcinoma – long-term results and literature review on QLQ-C30 and QLQ-PR25 results in published brachytherapy series
Source: Health Qual Life Outcomes. 2018 Jan 22;16:21. doi: 10.1186/s12955-018-0844-8 (PMC5778674; doi:10.1186/s12955-018-0844-8)
Supplement: Supplementary file 2 — Correlations between QLQ-PR25 scores and general quality of life. (DOCX 17 kb) [file 12955_2018_844_MOESM2_ESM.docx]

**Supplementary Table 2:** Correlations between QLQ-PR25 scores and general quality of life.

|  |  | **Age** | **QL** | **PRSAC** | **PRSFU** | **PRURI** | **PRBOW** | **PRHTR** | **PRAID** |
| --- | --- | --- | --- | --- | --- | --- | --- | --- | --- |
| **Age** | **Correlation Coefficient** | 1.000 | -.059 | -.288(*) | -.209 | .068 | -.090 | .071 | .484 |
|  | **Significance** | . | .604 | .013 | .235 | .549 | .449 | .544 | .094 |
|  | **N** | 80 | 80 | 74 | 34 | 79 | 73 | 76 | 13 |
| **QL** | **Correlation Coefficient** | -.059 | 1.000 | .319(**) | .251 | -.481(**) | -.345(**) | -.558(**) | -.622(*) |
|  | **Significance** | .604 | . | .006 | .152 | .000 | .003 | .000 | .023 |
|  | **N** | 80 | 80 | 74 | 34 | 79 | 73 | 76 | 13 |
| **PRSAC** | **Correlation Coefficient** | -.288(*) | .319(**) | 1.000 | .557(**) | -.351(**) | -.060 | -.304(**) | -.291 |
|  | **Significance** | .013 | .006 | . | .001 | .002 | .621 | .009 | .358 |
|  | **N** | 74 | 74 | 74 | 33 | 73 | 70 | 73 | 12 |
| **PRSFU** | **Correlation Coefficient** | -.209 | .251 | .557(**) | 1.000 | -.335 | -.257 | -.618(**) | .000 |
|  | **Significance** | .235 | .152 | .001 | . | .053 | .162 | .000 | 1.000 |
|  | **N** | 34 | 34 | 33 | 34 | 34 | 31 | 32 | 4 |
| **PRURI** | **Correlation Coefficient** | .068 | -.481(**) | -.351(**) | -.335 | 1.000 | .319(**) | .560(**) | .383 |
|  | **Significance** | .549 | .000 | .002 | .053 | . | .006 | .000 | .196 |
|  | **N** | 79 | 79 | 73 | 34 | 79 | 72 | 75 | 13 |
| **PRBOW** | **Correlation Coefficient** | -.090 | -.345(**) | -.060 | -.257 | .319(**) | 1.000 | .421(**) | .896(**) |
|  | **Significance** | .449 | .003 | .621 | .162 | .006 | . | .000 | .000 |
|  | **N** | 73 | 73 | 70 | 31 | 72 | 73 | 73 | 13 |
| **PRHTR** | **Correlation Coefficient** | .071 | -.558(**) | -.304(**) | -.618(**) | .560(**) | .421(**) | 1.000 | .779(**) |
|  | **Significance** | .544 | .000 | .009 | .000 | .000 | .000 | . | .002 |
|  | **N** | 76 | 76 | 73 | 32 | 75 | 73 | 76 | 13 |
| **PRAID** | **Correlation Coefficient** | .484 | -.622(*) | -.291 | .000 | .383 | .896(**) | .779(**) | 1.000 |
|  | **Significance** | .094 | .023 | .358 | 1.000 | .196 | .000 | .002 | . |
|  | **N** | 13 | 13 | 12 | 4 | 13 | 13 | 13 | 13 |

* Correlation is significant at the 0.05 level (2-tailed).

** Correlation is significant at the 0.01 level (2-tailed).
